# More about Leeches

**Published:** 1862-08

**Authors:** 


### MORE ABOUT LEECHES.

Our other half gave "*A Little Experience with Leeches*" in the last No. of the Register; we will add a little on the same subject. We keep leeches pretty much as Dr. W. does, though our course with them is about as follows: After they have been applied, we do nothing to make them disgorge, for the ordinary methods of treating them for that purpose are more or less injurious. They are frequently killed by the application of salt, as also by "stripping," though, perhaps, either may be employed with care, and not do much injury. But we think the better method is simply to let them digest the blood; it *does not* kill them, nor does it injure them. They will usually digest the blood in about ten days; during this time they should be kept in water, and the water changed every two or three days, and every time the change is made, the leech should be thoroughly washed through several waters, by stirring or whipping it round with a little stick with cotton rolled upon the end, so as to do no injury. After the leech is reduced to about his natural size, it is better to keep him in his native soil; this can be obtained of those who import or keep them for sale. Then, just before using, remove and put them in a jar of water and whip them, as already described, when they will become very active, and seem to be angry; then they will take hold, wherever applied, with great energy. We have leeches that we have kept a year, and that have been applied, perhaps, more than twenty times, that are now as vigorous and savage as any leeches we ever saw, and we would pit them against the best. We have had three or four get out and *run off*. T.

